# Genetic susceptibility to multiple sclerosis: interactions between conserved extended haplotypes of the MHC and other susceptibility regions

**DOI:** 10.1186/s12920-021-01018-6

**Published:** 2021-07-10

**Authors:** D. S. Goodin, P. Khankhanian, P. A. Gourraud, N. Vince

**Affiliations:** 1 Department of Neurology, University of California, UCSF MS Center, San Francisco 675 Nelson Rising Lane, Suite #221D, CA 94158 San Francisco, USA; 2 Center for Neuro-Engineering and Therapeutics, University of Pennsylvania, Philadelphia, PA USA; 3 Centre de Recherche en Transplantation Et Immunologie, UMR 1064, INSERM, Université de Nantes, Nantes, France; 4 Institut de Transplantation Urologie Néphrologie (ITUN), CHU Nantes, Nantes, France

**Keywords:** Genetic, Susceptibility, Multiple sclerosis, Risk, Additive, Multiplicative, Combination, Epidemiology

## Abstract

**Background:**

To study the accumulation of MS-risk resulting from different combinations of MS-associated conserved-extended-haplotypes (*CEHs*) of the *MHC* and three non-*MHC* “risk-haplotypes” nearby genes *EOMES, ZFP36L1,* and *CLEC16A*. Many haplotypes are MS-associated despite having population-frequencies exceeding the percentage of genetically-susceptible individuals. The basis of this frequency-disparity requires explanation.

**Methods:**

The *SNP*-data from the WTCCC was phased at the *MHC* and three non-*MHC* susceptibility-regions. *CEHs* at the *MHC* were classified into five haplotype*-*groups: (HLA-DRB1*15:01 ~ DQB1*06:02 ~ *a1*)-containing (*H* +); extended-risk (*ER*); all-protective (*AP*); neutral (*0*); and the single-*CEH* (*c1*). MS-associations for different “risk-combinations” at the *MHC* and other non-*MHC* “risk-loci” and the appropriateness of additive and multiplicative risk-accumulation models were assessed.

**Results:**

Different combinations of “risk-haplotypes” produce a final MS-risk closer to additive rather than multiplicative risk-models but neither model was consistent. Thus, (*H* +)-haplotypes had greater impact when combined with (*0*)-haplotypes than with (*H* +)-haplotypes, whereas, (*H* +)-haplotypes had greater impact when combined with a (*c1*)-haplotypes than with (*0*)-haplotypes. Similarly, risk-genotypes (*0,H* +), (*c1,H* +), (*H* + *,H* +) and (*0,c1*) were additive with risks from non-*MHC* risk-loci, whereas risk-genotypes (*ER,H* +) and (*AP,c1*) were unaffected.

**Conclusions:**

Genetic-susceptibility to MS is essential for MS to develop but actually developing MS depends heavily upon both an individual’s particular combination of “risk-haplotypes” and how these loci interact.

**Supplementary Information:**

The online version contains supplementary material available at 10.1186/s12920-021-01018-6.

## Background

The nature of susceptibility to multiple sclerosis (MS) is quite complex and involves both environmental and genetic factors [1–4]. Recently, considerable progress has been made in our understanding of the basis for “genetic susceptibility” in MS. Thus, to date, over 200 common risk variants (located in diverse, largely autosomal, genomic regions) have been identified as being MS-associated by genome-wide association screens (*GWAS*) using large arrays of single nucleotide polymorphisms (*SNPs*) scattered throughout the genome [5–14]. Despite this recent explosion in the number of identified MS-associated regions, however, certain alleles of the human leukocyte antigens (*HLA*) inside the major histocompatibility complex (*MHC*), known for decades, are still the main genetic contributors to this susceptibility [11, 15–22]. The importance of these new observations to our understanding of “genetic-susceptibility” in MS is tempered by the fact that any single *SNP* is generally the marker of a genomic region. Indeed, the presumptively associated (i.e., “candidate”) genes can be at a considerable genetic distance from the location of the *SNP* itself [12–14].

For example, we described an 11-*SNP* haplotype (*a1*), which spans 0.25 megabases (mb) of DNA surrounding *HLA-DRB1*, and which has the most significant association with MS of any *SNP* haplotype in the genome [23, 24]. Moreover, 99% of (*a1*) *SNP* haplotypes carry the HLA-DRB1*15:01 ~ HLA-DQB1*06:02 haplotype and, conversely, 99% of these *HLA-*haplotypes carry the (*a1*) *SNP* haplotype. Individually, each *SNP* is highly associated both with this particular *HLA-*haplotype and with MS, but for none of them is this association exclusive [26]. Thus, each of these *SNPs* is also found in association with other *HLA-*haplotypes [24, 26]. Consequently, even with the large number of *SNP*s now identified as being MS-associated [13, 14], any such association can only be viewed as simply tagging a relatively large genomic region; it cannot be used with confidence to identify any specific gene or to implicate any specific allele with respect to its role in causing, or contributing to, a genetic-susceptibility for MS.

We and others have reported that the *MHC* region is composed of a relatively small collection of highly conserved extended haplotypes (*CEHs*), stretching across all of the “classical” *HLA* genes (*HLA-A, HLA-C, HLA-B, HLA-DRB1,* and *HLA-DQB1*)—a distance spanning more than 2.7 mb of DNA [25, 26]. As shown in Additional file 1: Figure S4, this same basic population structure is also found in numerous other widely separated human populations around the world [25]. These *CEHs* seem to be under a strong selection pressure, presumably based upon favorable biological properties of the complete haplotype [26]. Lastly, this population structure is unlikely to be the result of a linkage disequilibrium caused by the founder effects of a small population migrating out of Africa and radiating throughout Eurasia and the Americas. Rather, the marked divergence of the *CEH* composition both among and between these different human groups, including Africans (Additional file 1: Tables S4a & S4b), indicates that this population structure must be due to local selection [26]. Consequently, “genetic-susceptibility” to MS is not likely to be attributable to any specific *HLA* allele but, rather, seems to depend upon the nature of each *CEH* [26]. For example, we have described a collection of *SNP*-haplotypes that are composed of unique combinations of the 11 *SNPs* (*rs2395173*; *rs2395174*; *rs3129871*; *rs7192*; *rs3129890*; *rs9268832*; *rs532098*; *rs17533090*; *rs2187668*; *rs1063355*; and *rs9275141*), and which span 0.25 *mb* of DNA surrounding the *HLA-DRB1* locus [23, 24]. Ten of these *SNPs* are within intergenic regions whereas *rs1063355* is within exon 5 of the *DQB1* gene. One of these SNP-haplotypes (*a1*) is strongly linked to HLA-DRB1*15:01 ~ HLA-DQB1*06:02, Thus, almost all of the *CEHs*, which contain the HLA-DRB1*15:01 ~ HLA-DQB1*06:02 haplotype, are linked to the *a1 SNP*-haplotype, and all of these are associated with an increased MS-risk, although the magnitude of the association varies significantly among the different *CEHs* [25]. Nevertheless, some rare haplotypes, which include the Class II motif of HLA-DRB1*15:01 ~ HLA-DQB1*06:02 but are not linked to (*a1*), seem not to carry any risk [26]. By contrast, haplotypes containing (*a1*), but not this Class II *HLA-*motif, still carry substantial risk [26]. Similarly, the disease risk for *CEHs* carrying HLA-DRB1*03:01 ~ HLA-DQB1*02:01 ~ *a2* differed considerably from *CEHs* carrying HLA-DRB1*03:01 ~ HLA-DQB1*02:01 ~ *a6* and*,* for the latter group of *CEHs*, the disease association varied widely depending upon the exact *CEH* composition (Additional file 1 Table S2). Finally, the *MHC* allele HLA-A*02:01 has been previously reported to be protective [27]. In the WTCCC, this allele was also found to be “protective” (*OR* = 0.69; p < 10^–29^), although, again, the association depends upon which *CEH* this allele resides rather than upon the presence of the HLA-A*02:01 allele itself (Additional file 1 Tables S1 & S2). These examples underscore the complex interactions that take place between the various *MHC* alleles/haplotypes and MS-risk.

However, in addition to the *MHC*, other “risk” loci are worth considering. Here we focus on three non-*MHC* “risk-regions” of interest, which are nearby genes: *EOMES*, (*Region d1*), a transcription factor specific related to T-cell differentiation; *ZFP36L1*, (*Region d2*), a transcription factor involved in cell activation; and *CLEC16A*, (*Region d3*), a cell surface protein whose family is involved in cell activation.

In the present manuscript, we explore these relationships and interactions between the different disease-associated *CEHs* in the *MHC* region and the other “risk” haplotypes elsewhere in the genome, in order to shed light on the nature of genetic susceptibility to MS. In addition, we evaluated the manner in which disease-risk is accumulated by the combination of one or more MS-risk factors in the same individual. There are two basic epidemiological models for this accumulation of disease-risk (see Additional file 1), which have been widely utilized—the so-called additive and multiplicative relative-risk (*RR*) models [28–32]. Often, however, actual epidemiological observations don’t fall neatly into one model or the other. In studies of the genetic susceptibility to MS, multiplicative risk models have generally been utilized [33–35], although this choice may not be appropriate in all circumstances (see Additional file 1 ).

Although, in this manuscript, we focus on the genetic aspects of MS pathogenesis, MS susceptibility is complex and involves genetic factors, environmental factors, and their interaction [1–4]. In fact, we have recently published an in-depth consideration of these issues as well as the important implications that studies of mono-zygotic, dizygotic twins, and non-twin siblings have regarding both genetic and environmental aspects susceptibility to MS [36]. Here, however, we will not consider further the important environmental aspects of MS pathogenesis.

## Methods

### Ethics statement

This research has been approved by the University of California, San Francisco's Institutional Review Board (IRB) has been conducted according to the principles expressed in the Declaration of Helsinki.

### Study participants

#### Wellcome trust case control consortium (WTCCC)

The WTCCC cohort included 18,872 controls and 11,376 cases with MS. The patients enrolled in the multinational WTCCC were of European or European-American ancestry. This cohort has been described previously [12–14]. For 380 controls and 232 cases the *SNP* data was incomplete. All of the *SNP* data was post-quality control [12]. Of the cases, the average age-of-onset was 32.3 years, 72.9% were women, and the mean Extended Disability Status Score (EDSS) was 3.9 [12]. The large majority (89%) of the cases had a relapsing–remitting onset [12]. The diagnosis of clinically definite MS was based upon international criteria [37–39]. Control subjects were composed of healthy individuals with European ancestry [12]. The protocol was approved by the ethical committees or institutional review boards at each of the participating centers. Informed consent was obtained from each study participant. The WTCCC granted data access for this study.

### Genotyping, and quality control

The WTCCC methods for genotyping and quality control have been described previously [12–14, 16, 18, 19]. Genotyping was performed at the Wellcome Trust Sanger Institute on the Illumina Infinium platform and case samples were genotyped using a customized Human660-Quad chip. Common controls were genotyped on a second customized Human1M-Duo chip (utilizing the same probes). This provided data on 441,547 autosomal SNPs scattered throughout the genome in both MS patients and control subjects after quality control. We used the the HIBAG method [40] to impute the identities of the five *HLA* alleles in the *MHC* region (*A, C, B, DRB1* and *DQB1*). Imputation methods, apart from HIBAG, such as the so-called ‘SNP2HLA’ method [41], have also been proposed. However, in a comparative study in European Americans, the HIBAG method had slightly better concordance rates and had slightly worse call rates compared to SNP2HLA [42]. Nevertheless, these differences were minimal, and both imputation methods seem equally good and the use of either is appropriate [42].

### Statistical methods

#### Phasing

**Fig. 1:**
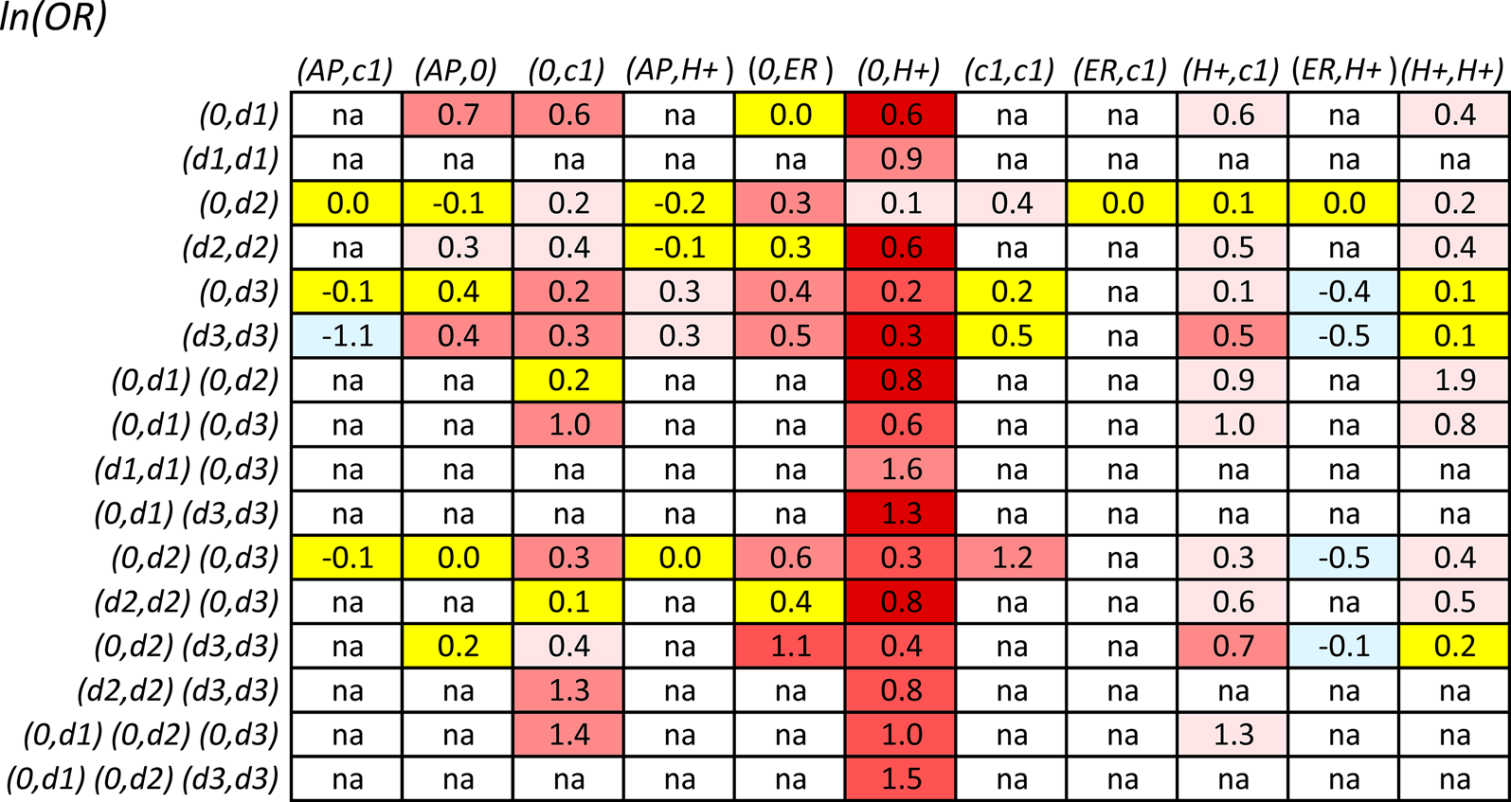
Natural logarithm of the odds ratios (*ORs*) for the different combinations of *MHC* and non-*MHC* genotypes. All *ORs* were calculated relative to a group consisting of the same *MHC* genotype and with the genotypes (*0,0*) at all non-MHC loci involved in the comparison – see text. The *MHC* genotypes, in order of increasing disease-risk (Fig. S2), are presented on the *x-axis* (as columns) and the genotypes at non-*MHC* loci are presented on the *y-axis* (as rows). The values for *ln*(*OR*) and the *z-scores* for each comparison are represented as numbers at the points of intersection of the column and row for any two haplotypes. Comparisons with a z-score (|*z|*< *1*) are shaded in yellow; comparisons with a z-score (*1* ≥*|z|*< *2*) are shaded in either pale blue (negative) or pale red (positive); comparisons with a z-score (*2* ≥ *z* < *3*) are shaded in light red; comparisons with a z-score (*3* ≥ *z* < *4*) are shaded in red; comparisons with a z-score (*z* ≥ *4*) are maroon. Specific combinations having marginal totals of less than15 total representations in the WTCCC are indicated by (*na*). Adjustment for multiple comparisons [45] only impacted the significance of *z-scores* less than 2.5

Alleles at each of five *HLA* loci (*HLA-A, HLA-C, HLA-B, HLA-DRB1* and *HLA-DQB1*) and the SNPs in the Class II region of the *DRB1* gene were phased using previously published probabilistic algorithms [23, 24, 43, 44]. SNP-haplotypes from 3 of the 102 non-MHC genomic regions, which had been identified previously as being significantly MS-associated, were also included in our analysis [24]. In our previous report, the MS-associated SNP haplotypes were numbered (arbitrarily) from 1 to 932. These three particular regions (arbitrarily labeled *d1*, *d2*, and *d3*) were selected based on their having a “risk” SNP-haplotype with 500 or more representations in the WTCCC dataset and also having the largest *ORs* for disease-association of any haplotype meeting this specification. The reason for choosing only three regions was that, when more regions were added, there was an insufficient number of observations to estimate the *ORs* for any of the possible higher order combinations [36]. Moreover, we only analyzed combinations having 15 or more representations in case or controls (combined) and of the 83 observations presented in Fig. 1, 18% had a marginal total of less than 25, 17% had a marginal total from 25 to 49 and 65% had a marginal total of 50 or more. Because the effect sizes were not able to be estimated in advance, it is possible that this approach may have considered some *ORs* to be non-significant because the sample sizes were too small. Nevertheless, for context, the percentage of comparisons reaching a nominal level of statistical significance (*p* < *0.05*) within each of these three groups was similar (27%, 29%, and 39%, respectively.

The three non-*MHC* regions that we analyzed were located at chromosomal locations 3p24.2, 14q24.1, and 16p13.13 and in the vicinity, respectively, of the genes *EOMES*, *ZFP36L1*, *CLEC16A* [13, 14]. Chromosome 3; Region 22 (*d1*) spanned 0.65 mb of DNA and the 11-SNP-haplotype (number 234) was used [24]. This region consists of 185 different SNP combinations, of which, 1,243 (2%) are the “risk” haplotype (01,100,000,100). Chromosome 14; Region 78 (*d2*) spanned 0.68 mb of DNA and the 3-SNP-haplotype (number 734) was used [24]. This region consists of 7 different SNP combinations, of which, 14,091 (23%) were the “risk” haplotype (111) Chromosome 16; Region 85 (*d3*) spanned 0.20 mb of DNA and the SNP-haplotypes (numbers 814, 818, and 822) were combined into a single 15-SNP haplotype [24]. This was done because each of these risk-haplotypes were adjacent to each other and because the individual risk SNP haplotypes were part of the same extended 15-SNP-haplotype. This region consists of 210 different SNP combinations, of which, 24,709 (41%) are the “risk” haplotype (000010000000000).

One might reasonably question why we used these three regions (chosen based on our WTCCC cohort) to assess the additive nature of the genetic risk when a much larger dataset has now been published and, when using this larger cohort might have led to a different choice for the regions of analysis [14]. However, when we requested access to this larger dataset, we were informed that we were “requesting individual level genotype data which is per definition sensitive data and therefore subject to varying levels of restrictions from all contributing parties within the IMGSC. Your request can therefore not be granted at this time”. Consequently, the current data is all that was available to us and, theoretically, the additive or multiplicative nature of the of the accumulation of genetic risk could be assessed using any 3 non-MHC risk-haplotypes. We chose these three regions because they were the ones that met our criteria and gave us the best statistical power (*see above*).

Also, it is important to note, that this earlier study [14], the authors looked at disease associations for more than 8 million *SNPs* and, controlling for both population stratification and genomic inflation, they identified (and replicated) disease associations for 233 loci (i.e., *SNPs*) that were confirmed to be MS-associated. Among these loci identified and, also, among those replicated in the earlier report from the WTCCC [13], were the 4 loci that we studied (i.e., the MHC together with the regions of the *EOMES*, *ZFP36L1*, & *CLEC16A* genes). By contrast, in the present manuscript, we are not looking to identify “true” disease associations. These are already well-established. Rather, we are looking at just four of these well-established and replicated loci, which are known, unequivocally, to be MS-associated to determine whether these loci (i.e., the associated SNP-haplotypes) exhibit either additive or multiplicative properties when forming different combinations of the risk variants.

#### Haplotype frequencies and association testing

Disease association tests, as measured by *ORs* and confidence intervals (*CIs*) comparing cases to controls, were calculated for each of the *CEHs* and each of the 3 non-*MHC* risk haplotypes either alone or in different combinations. The WTCCC data was considered in its entirety and not further stratified. MS-associated haplotypes were analyzed by grouping them into five categories of *CEHs* or haplotype groups (Additional file 1: Table S3), which consisted of: (1) (*H* +)-carrying *CEHs* (i.e. those containing the *HLA*-DRB1*15:01 ~ *HLA*-DQB1*06:02 ~ *a1* haplotype—Additional file 1: Table S1) other increased risk or “extended risk” (*ER*) *CEHs* (*c23, c27, c34, c46, c68, c81, c85, c96,* and *c107*—Additional file 1: Table S2); (3) decreased risk or “all protective” (*AP*) *CEHs* (*c5, c15, c18, c24, c30, c32, c51*, and *c73*—Additional file 1: Table S2); (4) all *CEHs* not in the (*H* +), *ER*, or *AP* groups, which were designated as (*0*) *CEHs*; and (5) the (*c1*) *CEH* by itself. We also explored “protective” groups, which either included only the (*c5*) *CEH* or excluded this *CEH*. However, these analyses are not presented because the findings were the same as when the *AP* group was analyzed as a whole. In many circumstances, an individual’s *MHC* genotype was specified by the haplotype combination that they possessed. For example, by this convention, an individual homozygous for (*H* +) would be characterized as the (*H* + *, H* +) *MHC* genotype. By contrast, a heterozygous individual would be characterized as having the (*H* + *, 0*), the (*H* + *, ER*), the (*H* + *, c1*), or the (*H* + *, AP*) *MHC* genotypes. In the principal analysis, all MS-associations were assessed compared to a reference group consisting of the (*0, 0*) *MHC* genotype. On occasion (e.g., Additional file 1), for notational simplicity, when using the (*AP, AP*) *MHC* genotype as a reference, this genotype was referred to as (AP*).

Disease associations for the risk *SNP*-haplotypes on Chromosomes 3, 14, and 16, were assessed compared to a reference group consisting of the (*0,0*) *MHC* genotype, and excluded individuals carrying their risk-haplotypes at these chromosomal locations. We designate (collectively) all non-risk-haplotypes at each of these chromosomal locations as the (*0*) haplotype at each locus.

The significance of the differences in *ORs* for disease association (comparing cases to controls) for any two haplotypes or genotypes was determined by *z-scores* calculated from the differences in the natural logarithm of the *ORs* such that:

$$z = [\ln (OR1) - \ln (OR2)]/\sqrt {\{ SE[\ln (OR1)]\} ^{2} + \{ SE[\ln (OR2)]\} ^{2} }$$

The Benjamini-Hochberg (BH) method was used to correct for multiple testing of the different possible haplotype combinations both within the *MHC* and also for combinations of the *MHC* with the other genetic regions [45]. This method was chosen rather than the Bonferroni method because the BH approach has been proven to be far less stringent than Bonferroni while still controlling the “Type I family-wise error rate” for multiple comparisons to be less than any desired α-level [45]. As discussed in Additional file 1, pair-wise comparisons of *ORs* are independent of the reference group chosen. The *MHC* genotype (*0, 0*) had the largest sample size of any (Additional file 1: Table S3) and, therefore, to maximize the statistical power to detect differences, the *ORs* used for pair-wise comparisons within the *MHC* were estimated relative to a reference group consisting of the (*0,0*) genotype at both the *MHC* and also at the any non-*MHC* locus included in the comparison. As noted in the *Discussion*, such a method eliminates the common reference group disease-risk to yield an estimate of the pairwise *RR*. Within the WTCCC cohort, we used a principal components (*PC*) analysis excluding *MHC SNPs* (Eigensoft) to correct the observations in Additional file 1: Tables S2 & S3 for the possible effects of population stratification, as well as regression analysis to correct for the possible effects of geographic heterogeneity [25]. These adjustments did not significantly alter any of the associations identified (Additional file 1: Tables S1 & S2).

#### Evaluating additive and multiplicative risk-models

The *ORs* for the *MHC* alleles (*H* + *, ER,* and *0*) were determined relative to the (*AP, AP*) or (AP*) reference group, which was assigned a value of (*R*_*b*_ = *R*_AP*_ = 1)—see Additional file. These observed *ORs* were used to estimate the *RRs* associated with each set of *MHC* alleles and, in turn, these *RRs* were used to test the additive and multiplicative risk-models for the accumulation of disease risk considering different allelic combinations at the MHC. Subsequently, using a reference group consisting of the (*0,0*) *MHC* genotype, we determined the *ORs* for susceptibility alleles in the three non-*MHC* susceptibility regions—(*d1*)*,* (*d2*) and (*d3*)*.* The (*0,0*) *MHC* genotype was chosen as the reference both because this was the largest haplotype group in the WTCCC and because there were too few representations of the (*AP, AP*) *MHC* genotype in the WTCCC dataset (Additional file 1: Table S3). Nevertheless, these observed *ORs* were mathematically converted into *ORs* relative to the (*AP, AP*) or (AP*) *MHC* genotype and these re-referenced *ORs*, together with the *ORs* observed for the different allelic combinations at the *MHC*, were used to estimate the *RRs* associated with each allelic combinations at these four genomic locations (the *MHC* plus the three non-*MHC* susceptibility regions). These estimated *RRs* were then used to test the additive and multiplicative risk-models for accumulation of disease risk considering the different allelic combinations at these four susceptibility regions. In all cases, only *ORs* estimated from combinations with ≥ 15 representations in the WTCCC were considered.

## Results

### The MHC

In the *HLA* region, there were 146 *CEHs*, which had 50 or more representations in the WTCCC dataset, and these accounted for 48% of the total number (59,884) of *CEHs* present. Information about 45 of these *CEHs*, which were previously found to have some relationship to MS susceptibility [24], is provided in Additional file 1: Tables S1 & S2. Considering the often low *CEH* frequencies in the WTCCC, we classified them (Additional file 1: Table S3) into five haplotype-groups: (1) (*H* +) *CEHs* (i.e., those containing the *HLA*-DRB1*15:01 ~ *HLA*-DQB1*06:02 ~ *a1* haplotype,—Additional file 1: Table S1); (2) other increased risk or “extended risk” (*ER*) *CEHs* (*c23, c27, c34, c46, c68, c81, c85, c96,* and *c107*—Additional file 1: Table S2); (3) decreased risk or “all protective” (*AP*) *CEHs* (*c5, c15, c18, c24, c30, c32, c51*, and *c73*—Additional file 1: Table S2); (4) the “neutral” group (*0*) consisting of all those *CEHs* which did not belong to the (*H* +), (*ER*), or (*AP*) groups; and (5) the (*c1*) *CEH* by itself. Each of these groups of *CEHs* seemed to be segregating independently and, in the control group, frequencies for each of the different combinations were, statistically, at their Hardy–Weinberg expectations. Considering all of the combinations of “risk” *CEHs* {relative to the (*0,0*) MHC genotype}, the (*H* +)-haplotypes accounted for 81% of the risk haplotypes in the control population and for approximately the same percentage of this risk in both men and women (80% and 82% respectively). Moreover, the likelihood of men in the control population possessing a “risk”-*CEH* combination (26%) was approximately the same as the likelihood in women (27%). Similarly, the likelihood of men in the control population possessing an *AP CEH* (9%) was approximately the same as the likelihood in women (8%).

The subset of individuals who don’t carry any (*H* +), *ER*, or *AP CEHs* at the *MHC* is referred to as the (*0,0*) *MH*C genotype. In Additional file 1: Figures S1, S2, & S3, all *ORs* are presented relative to this group. All of the (*H* +) *CEHs* with 50 or more representations were significantly associated with MS-risk (Additional file 1: Table S2), as were, collectively, (*H* +)-carrying *CEHs* with fewer than 50 representations in the WTCCC (Additional file 1: Figure S1). Moreover, assessing, collectively, only those (*H* +)-carrying *CEHs* that had a single representation in the WTCCC, the disease association is still highly significant and of similar magnitude to other (*H* +)-carrying *CEHs* (i.e., *OR* = *3.0; CI* = *2.7 − 3.4; p* < *10*^*−10*^). Consequently, the (*H* +) haplotype, by itself, seems to contribute to the disease susceptibility in an individual although, as shown in Additional file 1: Table S1, the magnitude of this effect varies among different (*H* +)-carrying *CEHs* [25].

In addition, we defined different “risk” *CEH* combinations as: (1) “single copy risk” [1 copy of any (*H* +) *CEH* or any *ER CEH*] plus one copy of a (*0*) *CEH*; and: (2) “double copy risk” [2 copies of any (*H* +) *CEH*, the (*c1*) *CEH*, or any *ER CEH*, or combinations of {(*H* +) + *ER*}*,* {(*H* +) + (*c1*)}, or {*ER* + (*c1*)}]. The different “protective” *CEH* combinations were defined similarly as: (1) “single copy protective” [1 copy of an *AP CEH*] plus one copy of a (*0*) *CEH*; and: (2) “double copy protective” [2 copies of an *AP CEH*].

The “single copy risk” of MS for either (*H* +) or *ER CEHs* in women (*OR* = *3.0; CI* = *2.8 − 3.2; p* < *10*^*−220*^) was greater (*z* = *2.4; p* = *0.009*) than the same risk in men (*OR* = *2.6; CI* = *2.4 − 2,8; p* < *10*^*−96*^). By contrast, the “double copy risk” of MS in women and men was about the same.

The impact on the phenotype of an individual in response to combining two *CEHs* at the *MHC* into a single genotype is shown in Additional file 1: Figures S1 & S2. For example, as has been well described previously [11, 15–22], combining two copies of the (*H* +)-haplotype into a single genotype markedly and significantly increases the disease association Additional file 1: Figure S1. Nevertheless, not all *(H* +)-carrying haplotypes have the same disease association [26]. For example, the *OR* for single copy carriers of the (*c2*) *CEH* is significantly greater (*z* = *3.4–4.8; p* = *10*^*–3*^*– 10*^*–6*^) than the *OR* for either single or double-copy carriers of the (*c3*) *CEH*.

Similarly, considering the *AP* group of *CEHs* (Additional file 1: Figure S2), we found a significant dose-dependent response such that possessing 2 copies of an *AP CEH* is significantly more “protective” than possessing only a single copy and, in addition, the magnitude of these “protective” effects is similar to the “risk” produced by *(H* +) *CEHs* (Additional file 1: Figure S2). Moreover, having an *AP CEH* significantly and substantially mitigates (*z* = *5.2; p* = *10*^*–7*^) the disease risk produced by single copies of (*c2*), (*c3*), or, more generally, any (*H* +)-haplotype (Additional file 1: Figure S2). A single copy of an *ER CEH* adds to the risk of a single copy of (*c2*), (*c3*), or any (*H* +) *CEH*, although it adds significantly less (*z* = *2.5; p* = *0.006*) than does a 2^nd^ copy of an (*H* +) *CEH* (Additional file 1: Figure S2). And, finally, the (*c1*) *CEH* acts in an apparently recessive manner with little, if any, disease risk (above the homozygous “neutral” genotype) produced by a single copy of this *CEH* (Additional file 1: Figure S2). Nevertheless, (and by contrast) a single copy of the (*c1*) haplotype adds significantly (*z* = *2.5–6.0; p* = *0.006–10*^*–9*^) to the disease risk produced by single copies of (*c2*), (*c3*), or, more generally, of any (*H* +)-haplotype (Additional file 1: Figure S2).

Additional file 1: Figure S3 presents the *ORs* for the various combinations of the non-*MHC* loci and, in general, as can be appreciated in the Figure, the disease risk for each of these regions seems to be dose dependent. Nevertheless, the increase in disease susceptibility that results from combining susceptibility genotypes at these three non-*MHC* loci with *MHC* genotypes is quite different for the different *MHC* configurations (Fig. 1). Thus, for example, the different combinations of these non-*MHC* “risk” haplotypes consistently increased the risk for (*0,H* +), (*H* + *,H* +), (*0,c1*), and (*H* + *,c1*) “risk” genotypes (Fig. 1). By contrast, for other “risk” genotypes such as (*AP,H* +) and (*ER,H* +) and for “protective” genotypes such as (*AP,0*) and (*AP,c1*), these other these non-*MHC* “risk” haplotypes seemed to contribute essentially nothing to the final risk (Fig. 1).

**Fig. 2:**
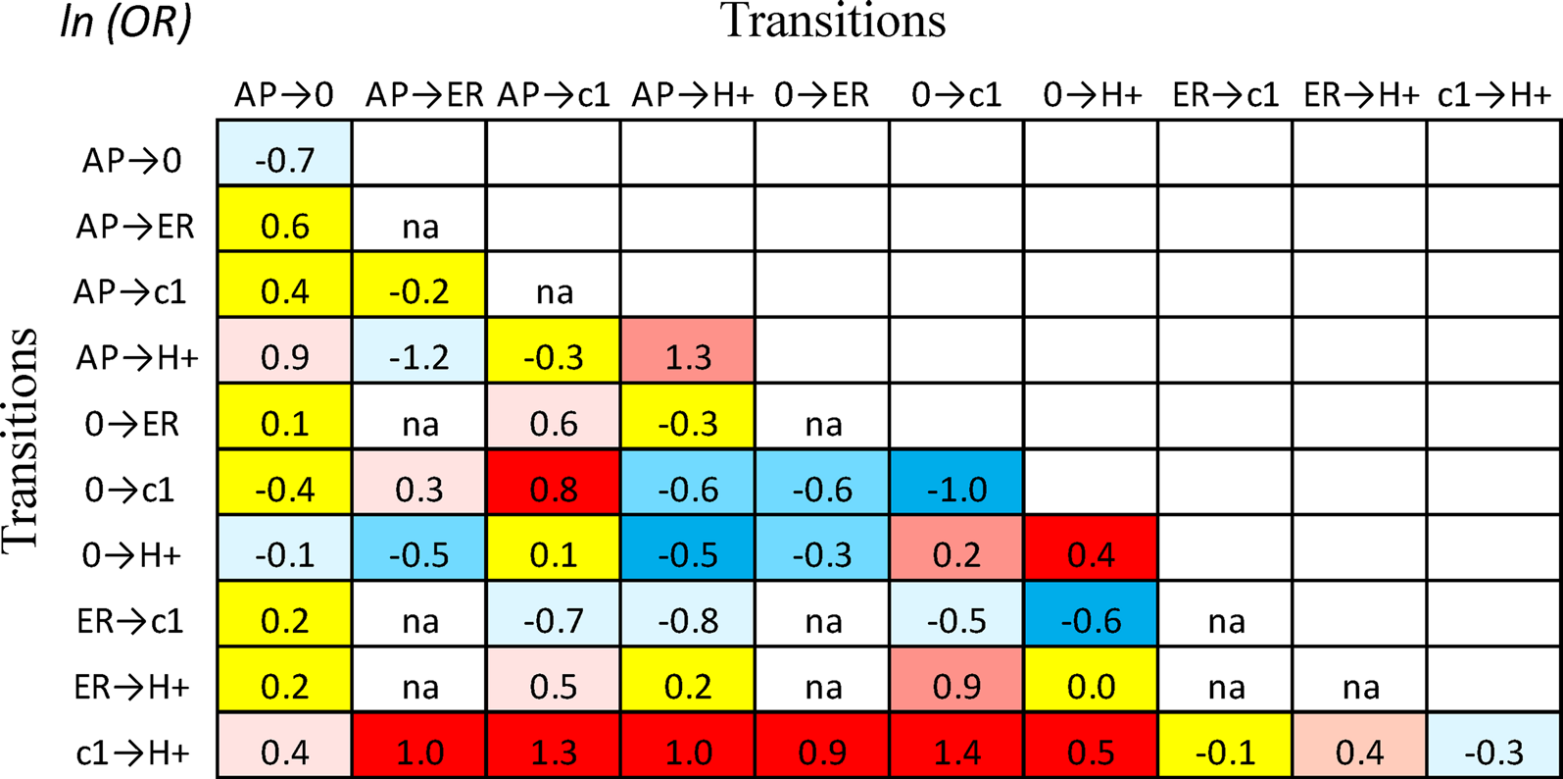
Lower triangular plots of the natural logarithm of the odds ratios (*ORs*) for replacing one *MHC* haplotype by another in different genotypic contexts. For example, at the point of intersection for $(0 \to H + )$ in the 7th column and $(c1 \to H + )$ in the in the 10th row, the value of (0.5) represents the log of the ratio: $\frac{{OR{\text{ for the transition: }}(c1,0) \to (c1,H + ){\text{ }} = {\text{ }}3.67}}{{OR{\text{ for the transition: }}(H + ,0) \to (H + ,H + ){\text{ }} = {\text{ }}2.17{\text{ }}}}$ or equivalently: $\frac{{OR{\text{ for the transition: }}(0,c1) \to (0,H + ){\text{ }} = {\text{ }}2.74{\text{ }}}}{{OR{\text{ for the transition: }}(H + ,c1) \to (H + ,H + ){\text{ }} = {\text{ }}1.62}}$. Positive numbers indicate that *OR* for the replacement transition (indicated by the column) using the 1st companion haplotype (indicated by 1st haplotype of the transition listed in the row) is greater than the same replacement transition using the 2nd companion haplotype (indicated by 2nd haplotype of the transition listed in the row). Conversely, a negative number indicates that the *OR* for the replacement transition using the 2nd companion haplotype is greater than it is using the 1st companion haplotype. Operationally, these replacement transitions are simply *ORs* comparing cases to controls for the two genotypes (e.g., $OR(c1,H + )/OR(c1,0) = 3.67$). Because of symmetry, this describes either of two, mathematically equivalent, replacement scenarios. The first compares the replacement of (*0*) by (*H* +) when the companion haplotype is (*c1*) to the same replacement when the companion is (*H* +). The second compares the replacement of (*c1*) by (*H* +) when the companion haplotype is (*0*) to the same replacement when the companion is (*H* +). Therefore, the interpretation for the meaning of the replacement transitions represented by the rows and column are interchangeable. These two, mathematically equivalent, replacement scenarios (i.e. transitions) can be depicted as follows:  . Only transitions for (*H* +), “extended risk” (*ER*), “all protective” (*AP*), (*c1*), and (*0*) haplotypes are shown. These transitions are indicated on both the *y-axis* (as rows) and the *x-axis* (as columns) and values of *ln*(*OR*) for each transition comparison are represented as numbers at the points of intersection of the column and row for any two transitions. Specific combinations having marginal totals of less than15 total representations in the WTCCC are indicated by (*na*). Comparisons of the *OR* for the numerator with the *OR* for the denominator, and which have an absolute z-score > 3.0, are shaded either dark blue (negative) or dark red (positive); comparisons having an absolute z-score = 2.0–3.0 are shaded either light blue (negative) or light red (positive); comparisons having an absolute z-score = 1.0–2.0 are shaded either pale blue (negative) or pale red (positive) yellow; comparisons having absolute z-scores < 1.0 are shaded in yellow. Adjustment for multiple comparisons [45] only impacted the significance of *z-scores* less than 2.5

Figure 2 shows the impact of replacing one *MHC* haplotype with another in different genotypic contexts. For example, replacing an (*0*)-haplotype with an (*H* +)-haplotype has a significantly greater impact when the companion haplotype is an (*0*)-haplotype compared to when the companion is an (*H* +)-haplotype (Fig. 2). Thus, comparing the (*0,H* +) genotype with the (*0,0*) genotype had an odds ratio of: (*OR*_*1*_ = *3.0*) whereas, comparing the (*H* + *,H* +) genotype with the (*H* + *,0*) genotype had an odds ratio of: (*OR*_*2*_ = *2.1*). These two *ORs* were significantly different from each other $(z = 4.7)$ and had a ratio of: $OR1/OR2 = 1.4$; and: $\ln (1.4) = 0.4$.

By contrast, replacing an (*0*)-haplotype with an (*H* +)-haplotype has a significantly smaller impact when the companion is an (*H* +)-haplotype compared to when the companion is a (*AP*)-haplotype (Fig. 2). Thus, comparing the (*H* + *,H* +) genotype with the (*H* + *,0*) genotype had an odds ratio of: (*OR* = *2.1*) whereas, comparing the (*AP,H* +) genotype with the (*AP,0*) genotype had an odds ratio of: (*OR* = *3.4*). These two *ORs* were significantly different from each other $(z = - 3.4)$ and had a ratio of: $OR1/OR2 = 0.6$; and: $\ln (0.6) = - 0.5$.

As can be appreciated from the figure, the impact of replacing one haplotype with another often depends considerably (and significantly) upon the nature of the companion haplotype, which, together with the haplotype being replaced, constitutes the *MHC* genotype (Fig. 2). This reflects the multiple haplotype-haplotype interactions that exist within the *MHC*. Indeed, if no such interactions were present, each of the comparisons provided in the figure would have an *OR* of ~ 1.0—i.e., *ln*(*OR*) = 0—i.e., they would be shaded in yellow (Fig. 2).

### Additive versus multiplicative risk

**Fig. 3:**
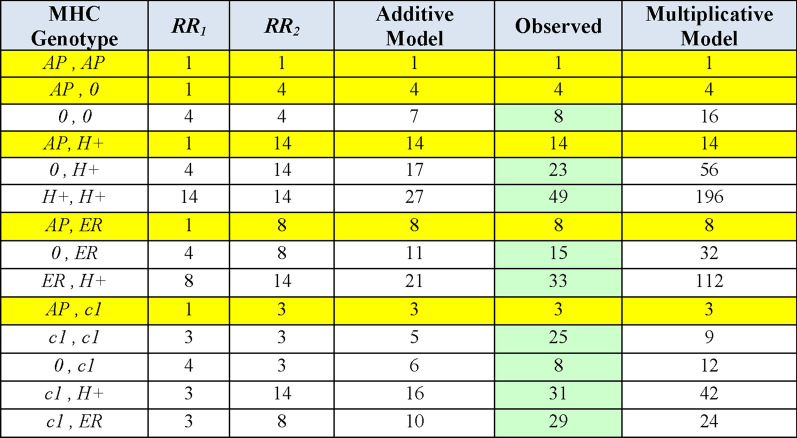
Conformity of the observed effect of combining different MHC haplotypes with an additive and a multiplicative model of combined risk. Yellow bands represent the definitional odds ratios (*ORs*) relative to a reference group consisting of the (*AP,AP*) or (AP*) genotype (i.e., as defined in the text: *R*_*b*_ = *R*_AP*_ = 1). With the exception of (*c1*), which seems to behave in a unusual fashion, the combination of other risk alleles produced, in general, a risk in between the two models, albeit closer to that predicted by the additive model. All combinations had, at least, 50 representations in the WTCCC and the green shading indicates the *ORs* actually observed. Cells with yellow shading in the “Observed” column also represents the *ORs* actually observed. However, in these yellow-highlighted cases, the *ORs* were used to approximate the relative risks (*RRs*), which, in turn, were used to assess whether the genotypes that are not yellow-highlighted conformed to the additive and multiplicative models (see “Methods” section)

**Fig. 4:**
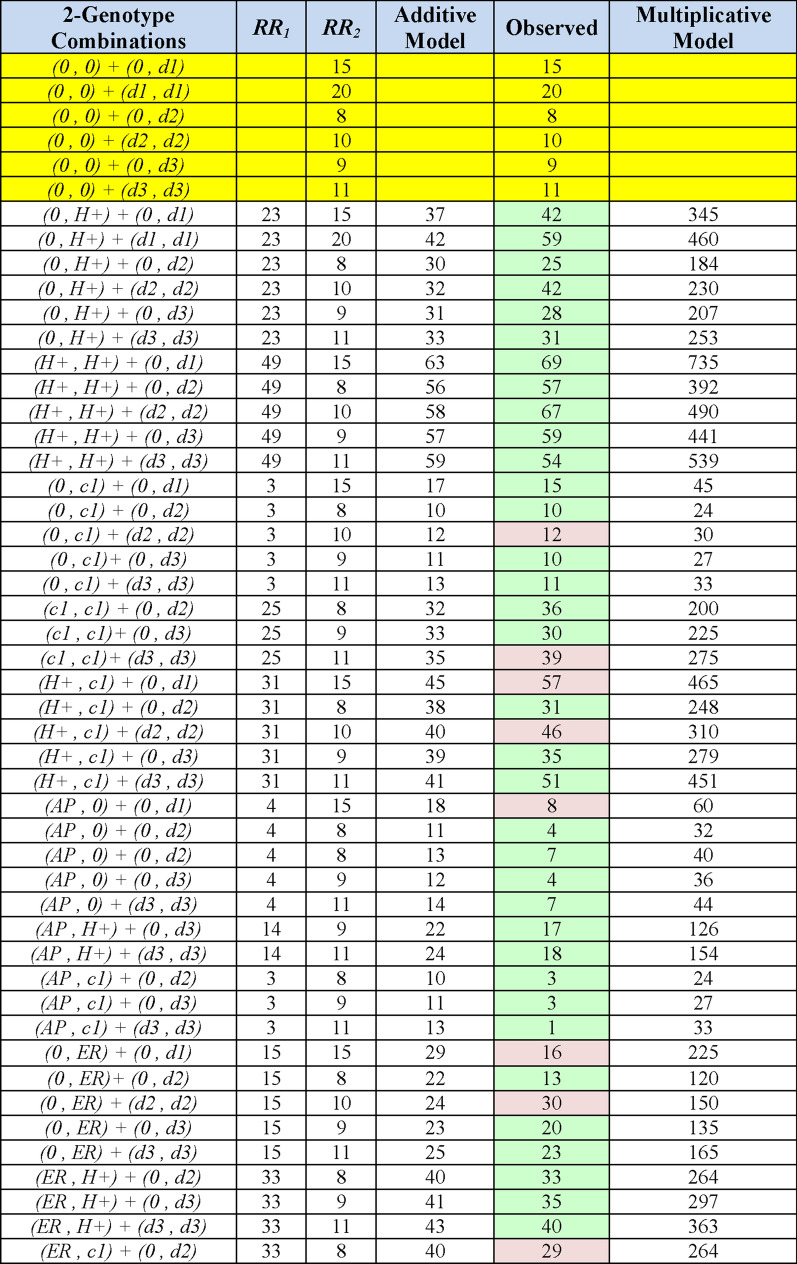
Conformity of the observed effect of combining different genotypes at the MHC and one susceptibility region with an additive and a multiplicative model of combined risk. The non-MHC susceptibility haplotypes are: (*d1*)*;* (*d2*)*;* and (*d3*)—see “Methods” section. Yellow bands, as in Fig. 4, represent the definitional *ORs* for different non-MHC genotypes actually observed, but which have been re-referenced to a group with the (*AP,AP*) MHC genotype. The *ORs* for all MHC genotypes are also those actually observed (Fig. 4). Only haplotype combinations with ≥ 15 or more representations in the WTCCC are shown. Combinations with fewer than 50representations are shaded in pink; combinations with at least 50 representations are shaded in green

**Fig. 5:**
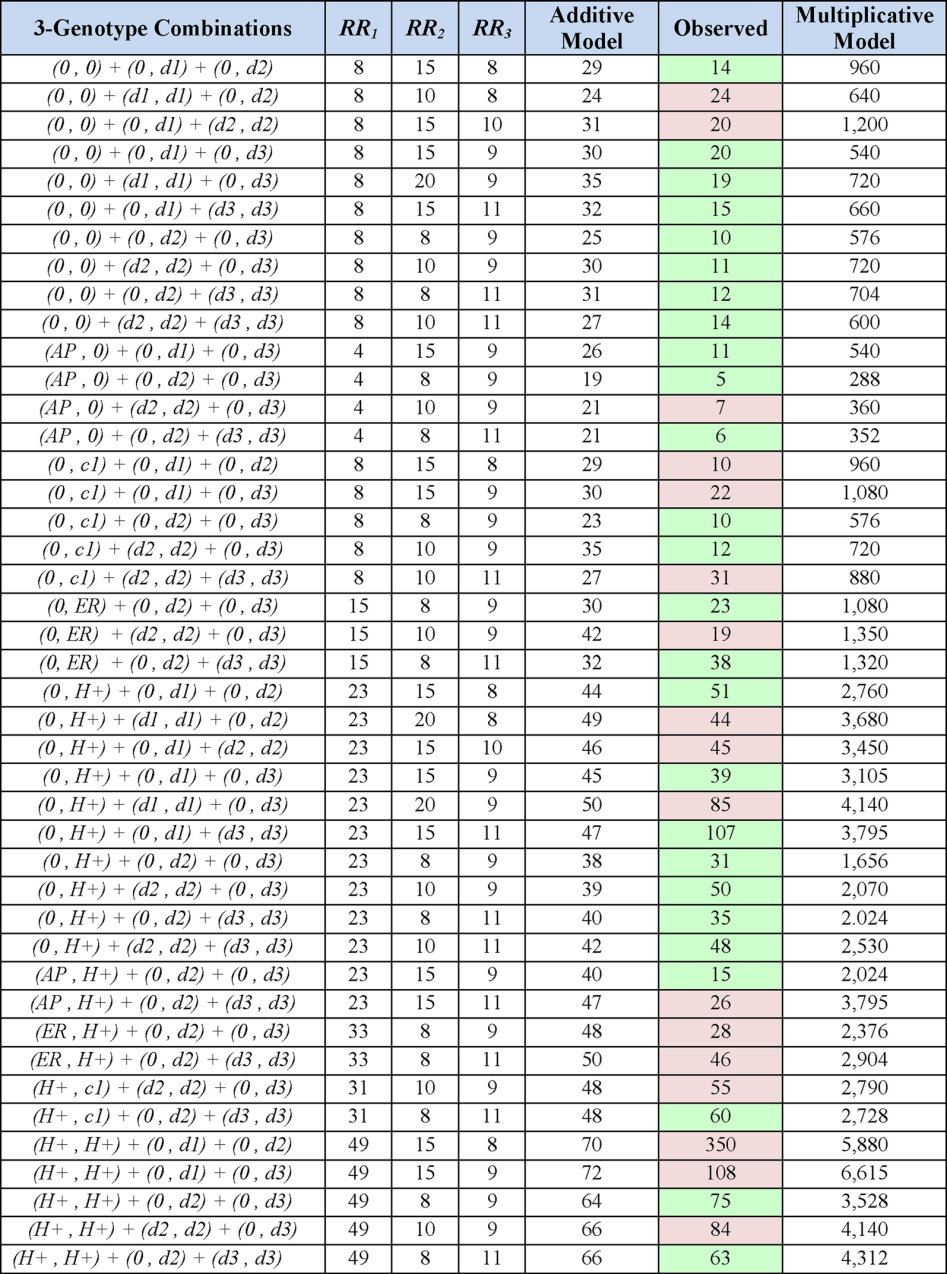
Conformity of the observed effect of combining different genotypes at the MHC and two susceptibility regions with an additive and a multiplicative model of combined risk. The non-MHC susceptibility haplotypes are: (*d1*)*;* (*d2*)*;* and (*d3*)—see “Methods” section. The *ORs* listed are those actually observed (Figs. 4, 5). Only haplotype combinations with ≥ 15 or more representations in the WTCCC are shown. Combinations with fewer than 50 representations are shaded in pink; combinations with at least 50 representations are shaded in green

**Fig. 6:**
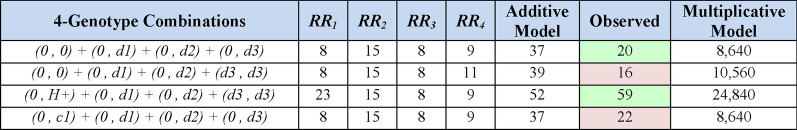
Conformity of the observed effect of combining different genotypes at the MHC and three susceptibility regions with an additive and a multiplicative model of combined risk. The non-MHC susceptibility haplotypes are: (*d1*)*;* (*d2*)*;* and (*d3*)—see “Methods” section. The *ORs* listed are those actually observed (Figs. 4, 5). Only haplotype combinations with ≥ 15 or more representations in the WTCCC are shown. Combinations with fewer than 50 representations are shaded in pink; combinations with at least 50 representations are shaded in green

Combinations of the 3 non-*MHC* susceptibility regions, together with different genotypes at the *MHC* are presented in Figs. 3, 4, 5, 6. In each of these Figures, the *ORs* are those derived from a comparison using, as a reference, the group having the (*AP, AP*) *MHC* genotype because this genotype had the lowest MS-risk of any we identified in the WTCCC (see Additional file 1). In all cases, the disease risk conferred by each genotype at each locus is estimated directly from the WTCCC observations (see “Methods” section). The expectations from the additive and multiplicative risk-models are then compared to the actual observations (Figs. 3, 4, 5, 6). In almost all cases, the additive model fits better with the actual observations than does the multiplicative model, especially as more “risk” loci are included in the combinations (Figs. 4, 5, 6). Nevertheless, neither model fits (or is approximated) consistently. For example, considering only those *MHC* “risk” genotypes that have a disease-risk exceeding that of the (*0,0*) *MHC* genotype, the actual disease-risk observed is, in general, greater than predicted by the additive model (Fig. 3). By contrast, considering 2-genotype combinations with only non-(*H* +) & non-*MHC* “risk” genotypes, the observed disease-risk is generally less than predicted by the additive model (Fig. 4). Such a trend becomes more noticeable (but not dramatically so) when more “risk” loci are included in the combinations (Figs. 5, 6). By contrast, the marked disparity with the multiplicative model becomes increasingly apparent as more “risk” loci are included in the analysis (Figs. 4, 5, 6).

## Discussion

The present findings provide considerable insight to the underpinnings of genetic susceptibility to MS and indicate that this susceptibility is complex as *MHC CEHs* groups can either be associated with an increased or decreased disease-risk. For example, the combination of 2 “risk” *CEHs* (*H* + or *ER*) results in an increased disease risk compared to a single copy of “risk” *CEH* alone (Additional file 1: Figures S1 & S2). Similarly, the combination to 2 “protective” *CEHs* (*AP*), results in a decreased disease risk compared to a single copy (Additional file 1: Figure S2). Finally, combining a “risk” *CEH* together with a “protective" *CEH* results in an intermediate disease risk compared with having a single copy of either type of *CEH* alone (Additional file 1: Figure S2). Nevertheless, there are exceptions to this general rule. Notably, a single copy of the (*c1*) *CEH* − the highest frequency *CEH* in both the WTCCC controls and other European populations [25, 26]—is associated with a negligible disease-risk (Additional file 1: Figure S2). By contrast, the disease-risk is significantly increased (*p* < *10*^*–11*^) in (*c*1,*c*1) individuals (Additional file 1: Figure S2), which suggests a recessive model. Nevertheless, a single (*c*1) *CEH* increases disease risk when combined with “risk” *CEHs* but not with “protective” *CEHs* (Additional file 1: Figure S2).

Our findings also have important implications with respect to additive and multiplicative causal models for the accumulation of genetic risk. In practice, some difficulties are encountered when trying to assess the appropriateness of either model. First, in a case–control studies (such as the WTCCC), because the incidence of the disease is not assessed (as it would be in a prospective cohort study), the actual *RRs* cannot be determined [46]. However, for a rare disease such as MS {e.g., where: $P(MS) \approx 0.003$}, the *ORs* and the *RRs* are almost identical [46] and, thus, can be used interchangeably. Second, selecting an appropriate reference group for calculating the *RRs* is crucial (see Additional file). This choice will influence how well the observations fit into one or another of these risk models. As discussed further in the Additional file 1:, the theoretical underpinnings for both the additive and multiplicative models arise from the same underlying probability assumptions [28–31] and are predicated on the notion that MS-risk for the different potential “risk-factors” is as great or greater than the “risk” in the reference group (Additional file 1). This requires identifying the reference group with the lowest MS-risk of any. In MS, however, because more than 93% of the population has no MS-risk whatsoever [36], the choice of this group is not possible due to the fact that the *RRs* become infinite or undefined (Additional file 1). In this situation, perhaps, using a reference group having a minimal (but non-zero) MS-risk, could permit the evaluation (approximately) of whether either of these two models fits with the data (Additional file 1). In the WTCCC, the (*AP, AP*) or (AP*) *MHC* genotype has the least MS-association [*OR* = 0.13 relative to the (*0,0*) *MHC* genotype] of any that we identified (Additional file 1: Figure S2). Therefore, this group was used to assess the appropriateness of the additive and multiplicative disease-risk models. The combination of 2 *CEHs* into a single *MHC* genotype, with the exceptions of (*c1*), produced an effect in between the two causal models (Fig. 3). By contrast, for (*c1,c1*) and (*c1,ER*) genotypes, the observations exceed the expectations of both models (Fig. 3).

When the other non-*MHC* “risk” loci are included in the analysis, the observations are closer to the additive model and even more when additional loci are included (Figs. 3, 4, 5, 6). Thus, the estimates from a multiplicative model ultimately exceed observations by 1–2 orders of magnitude (Figs. 3, 4, 5, 6). This observation is consistent with our previous observation that the distribution of penetrance values within the general population does not follow a lognormal distribution—i.e., the distribution expected for a multiplicative mode [3]. Similarly, as discussed further in Additional file 1, the multiplicative model breaks down as the disease-risk in the reference group becomes progressively lower. Consequently, based upon both theoretical considerations and observation, a multiplicative model for the accumulation of genetic risk in MS seems to be inappropriate.

The additive model, in general, performed better in these circumstances (Figs. 3, 4, 5, 6). Nevertheless, it does not explain perfectly the accumulation of genetic risk in MS. First, (*c1*)-containing *CEH* genotypes consistently exceed the additive expectations (Fig. 3). Second, effect of a given *MHC* haplotype is dependent on its companion *MHC* haplotype in a genotype (Fig. 3). Third, the effect of the 3 non-*MH*C “risk” haplotypes is not consistent across all *MHC* genotypes (Fig. 4). And fourth, when more loci are included in the analysis, the observations become increasingly less than what is predicted by the additive model (Figs. 3, 4, 5, 6). Taken together, these observations suggest that the accumulation of genetic risk from these “susceptibility loci” is inconsistent with both models. Rather, the magnitude of any change in disease-risk associated with the inclusion of additional “susceptibility loci” seems to depend upon the exact state at each “risk-locus” and on the interaction across all loci. Such a conclusion is also consistent with theoretical considerations [36].

The *MHC* is known to have a remarkable diversity [27] although the individual *HLA*-alleles occur as part of linked haplotypes or *CEHs* (Additional file 1). Even though some *CEHs* share common features, such as carrying the (*H* +)*-*haplotype, the degree of association with MS varies depending upon the exact *CEH* considered (Additional file 1: Tables S1 & S2). For example, both (*c2*) and (*c3*) *CEHs* carry the (*H* +)-haplotype, but their MS-association differed significantly (*z* = *4.8; p* < *10*^*–6*^). It might be tempting to attribute this difference to (*c3*) carrying the potentially “protective” HLA-A*02:01 allele (S2 File; Table S2). However, other HLA-A*02:01 and (*H* +) carrying *CEHs* (e.g., *c50, c58*, and *c139*) do not seem to be similarly protected (S1 File; Table S2). Finally, each identified *CEH* probably represents a diverse set of *CEHs*. Thus, because the genomic region from *HLA-A* to *HLA-DQB1* (spanning ~ 3 mb of DNA) is quite “gene-dense”, each of the *CEHs* that we defined, almost certainly, represent groups of *CEHs*, which carry many other linked variants of other genes.

Although the other non-*MHC* “risk” regions used in this analysis are likely to be less variable than the *MHC*, these regions span large amounts of DNA (200–680 kb) and they generally have hundreds of highly conserved *SNP*-haplotypes across each region. Moreover, even though authors sometimes identify specific genes as being MS-associated [13, 14], the truth is that we have no basis for deciding which gene or genes within a region are responsible for the association. We cannot exclude the possibility that, within these regions, as within the *MHC*, there might exist “risk” or “protective” alleles interacting with each other. If so, the likelihood that any simple probability model (either additive or multiplicative) will adequately describe genetic susceptibility to MS seems quite remote.

However, such complexity fits well with the nature of genetic susceptibility developed elsewhere [36]. Thus, more than 95% of the population has no chance of getting MS, regardless of what environmental experiences they have [36]. Moreover, even though the Class II (*H* +) haplotype is, by far, the strongest, and most significant, MS-associated genetic factor of any in the genome and has been known for over a half a century [11, 15–22, 26], only a small fraction (< 20%) of (*H* +) carriers have any chance getting MS [36]. This observation indicates that at least with respect to the (*H* +) haplotype, genetic susceptibility to MS requires the combined effects of different genes. Also, of the original 102 non-*MHC* “risk” loci identified by the WTCCC [13], only specific combinations increase the probability of being a member of the genetically susceptible subset [36]. Nevertheless, the combinations that make this membership more likely are quite heterogeneous, and among genetically susceptible individuals, only a small fraction share even the same 4-locus genetic combination [36]. This conclusion also supports the notion that genetic susceptibility to MS is largely idiosyncratic. Despite the need to be genetically susceptible in order for a person to develop MS, environmental factors (e.g., EBV infection and vitamin D deficiency) and stochastic factors are also critical components of disease-pathogenesis [36, 47–51]. Regardless of the identity of each factor, its role in pathogenesis, or when, during a person’s life, it acts, it seems clear that, collectively, these environmental and stochastic events are essential determinants of whether the disease will develop in any individual [36].

In the study of human genetics there has been a long-running debate between the so-called “common-disease, common variant” and the “common-disease, rare variant” hypotheses [52]. Nevertheless, with our improved genetic sophistication, it has become increasingly clear that, in different specific circumstances, either (or both, or neither) hypotheses could be operative [52]. In fact, our observations also support this notion. For example, on the one hand, all of the *MHC CEH* combinations, which impact genetic susceptibility to MS, are quite rare. None has a population frequency in controls of more than 6.2% and the large majority of them have population frequencies well below 1% (Additional file 1: Tables S1 & S2). On the other hand, considered collectively, those *CEH* combinations, which include the Class II (*H* +)-haplotype, have a WTCCC Control population frequency of 23%. Indeed, this particular haplotype-group is the most prevalent (and, therefore, the most highly selected) of all such Class II haplotype combinations in northern American and European populations [26]. Consequently, the most prevalent, and therefore the most highly selected [26], *CEHs* are also those that are associated with the highest MS-risk (Additional file 1: Tables S1 & S2). Thus, it is clear that these particular *CEHs* must come with both adaptive and deleterious consequences for the individual. In addition, although the *CEH* composition differs markedly among long-separated human populations (Additional file 1: Tables S4a & S4b), as shown in Additional file 1: Figure S4, specific *CEHs* are still being strongly selected in each of them [26]. Consequently, the benefits of the adaptive features of these *CEHs* must outweigh the risk of any deleterious ones. Obviously, for circumstances, either in which the risk of MS is small or in which MS has little impact on an individual’s eventual number of surviving children, even a modest advantage in favor of a specific *CEH* might still cause it to be selected. In this regard, a recent French study estimated that women with MS had 31% fewer children than their contemporary controls [53]. If this observation is correct, it suggests that there is a strong selective disadvantage to having MS. Therefore, the explanation for the benefits of these MS-associated *CEHs* outweighing the risks is likely to lie in an individual’s low risk of MS rather than the disease having little impact on their fertility. Based on our observations, this seems likely to be the case. Thus, because natural selection can only select against those genotypes, which actually carry risk (relative to other genotypes), both the fact that so few individuals are susceptible and the fact that so few of the individuals who are susceptible ever develop MS [36], makes such a favorable tradeoff between adaptive and deleterious features considerably more likely to occur.

## Conclusions

Comparing the different combinations of “risk” haplotype-groups to that of the final MS-risk demonstrates that an additive risk model is considerably more likely than a multiplicative risk-model. Nevertheless, even the additive-model is inconsistent for combinations of the four loci considered in this manuscript. For example, (*H* +)-haplotypes have a significantly greater impact when combined with (*0*)-haplotypes than they have when combined with other (*H* +)-haplotypes. By contrast, (*H* +)-haplotypes have greater impact when combined with a (*c1*)-haplotype than they have when combined with (*0*)-haplotypes. Similarly, risk-genotypes (*0,H* +), (*c1,H* +), (*H* + *,H* +) and (*0,c1*) were additive with risks from non-*MHC* risk-loci, whereas risk-genotypes (*ER,H* +) and (*AP,c1*) were unaffected by similar combinations.

Genetic susceptibility to MS is very uncommon in the population and, yet such susceptibility is essential for MS to develop [36]. How likely MS is to develop depends heavily upon both an individual’s particular combination of genetic “risk-loci” and also how these different loci interact with each other to make genetic-susceptibility more likely in that individual [36]. In addition, a person’s environmental experience, and stochastic processes play important roles in determining whether or not MS actually develops in a susceptible individual [36].

## Supplementary Information

**Additional file 1:** This Supplemental File describes composition of the *CEHs* found in the WTCCC dataset as well as their individual relationships to MS susceptibility, the impact on susceptibility of various combinations either of *CEHs* at the *MHC* or of the non-*MHC* loci and, finally, how this *CEH* composition differs between populations around the world. Furthermore, this file considers the theoretical underpinnings for the commonly used additive and multiplicative Models for the accumulation of disease “risk” with increasing number of “risk haplotypes” being present in an individual’s genotype.

## Data Availability

The Wellcome Trust Case Control Consortium (WTCCC) granted data access for this study. The data is available by request to the authors of the paper from which the WTCCC dataset is taken [12].

## Abbreviations

MHC: Major histocompatibility complex
HLA: Human leukocyte antigen
CEH: Conserved extended haplotype
ER: Extended risk
AP: All protective
SNP: Single nucleotide polymorphism
H +: The haplotype HLA-DRB1*15:01 ~ HLA-DQB1*06:02 ~ a1
EBV: Epstein Barr virus
OR: Odds ratio
WTCCC: Wellcome Trust Case Control Consortium
BH: Benjamini-Hochberg
MS: Multiple sclerosis
