# Supplementary material for: Genetic susceptibility to multiple sclerosis: interactions between conserved extended haplotypes of the MHC and other susceptibility regions
Source: BMC Med Genomics. 2021 Jul 10;14:183. doi: 10.1186/s12920-021-01018-6 (PMC8272333; doi:10.1186/s12920-021-01018-6)
Supplement: Supplementary file 1 — Additional file 1: This Supplemental File describes composition of the CEHs found in the WTCCC dataset as well as their individual relationships to MS susceptibility, the impact on susceptibility of various combinations either of CEHs at the MHC or of the non-MHC loci and, finally, how this CEH composition differs between populations around the world. Furthermore, this file considers the theoretical underpinnings for the commonly used additive and multiplicative Models for the accumulation of disease “risk” with increasing number of “risk haplotypes” being present in an individual’s genotype. [file 12920_2021_1018_MOESM1_ESM.pdf]

Supplemental Material

CEHs in the MHC and their Designations……………..…………………………. p.2

Relative Risk Models in MS.………………….……….………………………....… p.3

Risk in the Reference Population (R_b).………………….……..………………....… p.4

Supplemental Table S1 – (H+) CEHs……..……..………….……………......…....… p.8

Supplemental Table S2 – non-(H+) CEHs..……………………………………… p.9

Supplemental Table S3 – Summary Data; all CEHs ………..……….…………… p.10

Supplemental Table S4 – CEHs in World Populations……….……...……………… p.11

Supplemental Figure S1 – (H+) CEH combinations ……………......………………… p.12

Supplemental Figure S2 – all CEH combinations ……………………………………… p.13

Supplemental Figure S3 – non-MHC combinations …………………….……………… p.14

Supplemental Figure S4 – CEHs in World Populations…………………….……… p.15

CEHs in the MHC and their Designations

In the WTCCC population, there were 29 *HLA-A* alleles, 29 *HLA-C* alleles, 55 *HLA-B* alleles, 35 *HLA-DRB1* alleles, and 16 *HLA-DQB1* alleles. However, these *HLA*-alleles do not exist in isolation but, rather, occur as linked haplotypes, of which there are 10,078 unique *CEHs* in the WTCCC. Of these, only 810 account for 71% of all the *CEHs* (59,884) present in the WTCCC dataset [26]. In addition, 146 *CEHs* had 50 or more representations in the WTCCC dataset and these accounted for 48% of the *CEHs* present. Information on 45 of these *CEHs*, which were found in our previous study [24] to have some relationship to MS susceptibility, is provided in the (*Tables S1 & S2, below*). Of these, only four *CEHs* (*c1, c2, c3*, and *c5*) had a sufficient number of observations to assess the MS-risk of either homozygous combinations or combinations with each other. The *CEH*s of the WTCCC were divided into five haplotype groups: 1) (*H+*) *CEHs* (i.e., containing the *HLA-DRB1*15:01~HLA-DQB1*06:02~a1* haplotype; *see Tables S1 & S3, below*); 2) other increased risk or "extended risk" (*ER*) *CEHs* (*c23, c27, c34, c46, c68, c81, c85, c96,* and *c107*), as shown in *Tables S2 & S3* (*see below*); 3) decreased risk or "all protective" (*AP*) *CEHs* (*c5, c15, c18, c24, c30, c32, c51*, and *c73*), as shown in *Tables S2 & S3* (*see below*); 4) the "zero" group (*0*) consisting of all those CEHs which did not belong to the (*H+*), (*ER*), or (*AP*) groups; and 5) the (*c1*) *CEH* by itself (*see Table S3*). Each of these groups of *CEHs* seemed to be segregating independently and, in the control group, frequencies for each of the different combinations were, statistically, at their Hardy-Weinberg expectations.

In *Supplemental Figures S1, S2, & S3*, all *ORs* are presented relative to the (*0,0*) *MHC* genotype. The impact on the phenotype of an individual in response to combining two *CEHs* at the *MHC* into a single genotype is shown in *Supplemental Figures S1 & S2*. For example, as has been well described previously [11,15-22], combining two copies of the (*H+*)-haplotype into a single genotype markedly and significantly increases the disease association (*Supplemental Figure S1*). Nevertheless, not all *(H+)*-carrying haplotypes have the same disease association [26]. For example, the *OR* for single copy carriers of the (*c2*) *CEH* is significantly greater ($z=3.4–4.8; p=10^{-3}– 10^{-6}$) than the *OR* for either single or double-copy carriers of the (*c3*) *CEH* (*Supplemental Figure S2*). *Supplemental Fig S3* (*File S1*) presents the *ORs* for the various combinations of the non-*MHC* loci and, in general, as can be appreciated in the Figure, the disease risk for each of these regions seems to be dose dependent.

Human populations from many diverse parts of the world [25] are characterized by having a very small number of frequent *CEHs* and a large number rare (or novel) haplotypes (*Supplemental Fig S1*). Although native Africans have greater *CEH* diversity than the other

populations, even native Africans have this structure. That this population structure is due to selection (and not to a linkage disequilibrium caused by the founder effects of a small population migrating out of Africa and radiating throughout Eurasia and the Americas) is indicated by the fact that this distribution is markedly different from that expected in randomly distributed populations (*Supplemental Fig S1 – grey line*) and by the fact that these derivative populations are distinct both from one another and from their African forbearers (*Tables S4a & S4b, below*).

Relative Risk Models in MS

There are two basic epidemiological models for the accumulation of disease-risk, which have been widely utilized – the so-called additive and multiplicative risk models [55-59]. Nevertheless, actual epidemiological circumstances often don't fall neatly into one model or the other. Indeed, the same set of underlying probability assumptions can be used to approximate either an additive or a multiplicative risk model [56]. The difference depends upon the definition of the term "no interaction" between the risk factors [55-59]. In studies of the "genetic-susceptibility" to MS, multiplicative risk models have generally been utilized [60-62], although these models may not be appropriate in all circumstances. To illustrate some of the issues involved, we will consider two independent risk-factors (*A* and *B*), which are either causal or contributory to MS (or MS-susceptibility), as well as other background events (*b*), which can lead to MS in the absence of both (*A*) and (*B*). Defining (*A*−) and (*B*−) to represent the absence of each respective risk-factor, this background risk (R_b) can be expressed as:

$$R_b = P(MS \mid A-, B-)$$

The additive risk model arises naturally [55-59] when either:

$$P(A,B \mid MS) = P(A,B,MS) = 0$$

or: $$P(A,B,MS) = P(A,B) * P(MS \mid A,B-) + P(A,B) * P(MS \mid A-,B)$$

Such circumstances might occur when the two factors are mutually exclusive or when each factor makes the same contribution to the disease-likelihood, regardless of whether or not the other factor is also present. In the former circumstance, in which the sets (*A,MS*) and (*B,MS*) are disjoint, the relative risk (i.e., relative to R_b) for both factors (RR_{AB}) is related to the sum of the relative risks for the two factors considered separately (RR_A and RR_B). Specifically, under the former (disjoint) condition, it can be shown [54,55] that:

$$RR_{AB} = RR_A + RR_B - 1$$

In the latter circumstance, the combined risk is also a sum of risks although, in this case,

the relevant probabilities (risks) are:

$$P(A', MS) = P(A) * P(MS \mid A, B-) \leq P(A, MS)$$

and: $$P(B', MS) = P(B) * P(MS \mid A-, B) \leq P(B, MS)$$

such that: $$RR_{AB} = RR_{A'} + RR_{B'} - 1 \leq RR_{A} + RR_{B} - 1 .$$

By contrast, the multiplicative risk model, which is often assumed [60-62], arises naturally in circumstances where the two factors are conditionally independent such that:

$$P(A, B \mid MS) = P(A \mid MS) * P(B \mid MS)$$

Such circumstances might occur when the two factors affect different stages of a causal chain leading to the disease or when they otherwise contribute to producing disease in a conditionally independent manner. In these cases:

$$RR_{AB} = RR_{A} * RR_{B}$$

Each of these models, can easily be generalized to any number of risk factors. Thus, for example, with a 3rd risk factor (C), these equations become:

$$RR_{ABC} = RR_{A} + RR_{B} + RR_{C} - 2; \quad \text{and:} \quad RR_{ABC} = RR_{A} * RR_{B} * RR_{C}$$

If the two "risk" factors are alleles (or *CEHs*) at the same genomic location, then the risk models would need to include possibilities for both a so-called "dominant" and a "recessive" risk.

The "dominant" model would be:

$$RR_{AA} = RR_{AB} = RR_{A} > RR_{BB} \quad \text{or:} \quad RR_{BB} = RR_{BA} = RR_{B} > RR_{AA}$$

and the "recessive" model (for: $X \neq A$), compared to a reference ($A-, B-$), would be:

$$RR_{AX} = 1 \quad \text{and:} \quad RR_{AA} > 1$$

In a case-control studies (such as ours), because the incidence of the disease is not assessed (as it would be in a prospective cohort study), the actual *RRs* cannot be determined [63]. However, for a rare disease such as MS {e.g., where: $P(MS) \approx 0.003$}, the *ORs* and the *RRs* are almost identical [63] and, thus, can be used interchangeably.

Risk in the Reference Population (R_b).

Theoretical derivations for both the additive and multiplicative models arise from the same underlying probability assumptions [55-58], which are predicated on the relative risks being as great or greater than the "baseline" risk in the reference group (b). However, not all genetic factors increase risk and, in fact, in our analysis of MS risk in the WTCCC, of the 932 MS-associated non-*MHC* haplotypes reported previously, 607 were "protective" relative to a reference group, which lacked the specific haplotype being considered [24]. In any such analysis,

the composition of the "reference group" is, by definition, different for each *OR* calculated in this manner. Even using a single reference group, which excludes individuals who have certain "high-risk" haplotypes, some *CEHs* are still found to be "protective" (e.g., *Tables S1 & S2*, *below*).

However, this is simply a matter of definition. Thus, any observed "protective" impact of group (*A*) compared to a reference group (*B*) is equivalent to the "risk" impact of group (*B*) compared to a reference group (*A*). Moreover, importantly, although the relationship *between* any two *RRs* for different factors (i.e., the *RR* ratio) is independent of the reference group (*b*) chosen, the anticipated risk from combining these factors using these one of these two risk models is not.

To illustrate this, we will define the following relationships:

$$RR_A = R_A / R_b$$
$$RR_B = R_B / R_b$$
$$RR_A / RR_B = R_A / R_B$$
$$RR_A * RR_B = R_A * R_B / R_b^2$$
$$RR_A + RR_B - 1 = (R_A + R_B) / R_b - 1$$
$$RR_{AB} = R_{AB} / R_b$$

Notably, both the additive and multiplicative models:

$$RR_{AB} = RR_A * RR_B \quad \text{or:} \quad RR_{AB} = RR_A + RR_B - 1$$

only hold exactly when: $R_b = 1$

However, this is simply a matter of convention. For example, given an "*original*" reference population (*b1*), let us assume that the observed values for the "*original*" *RRs* follow one of these two relationships exactly.

Then, either: $RR_{AB}^{orig} = R_{AB} / R_{b1} = RR_A^{orig} * RR_B^{orig} = R_A * R_B / R_{b1}^2 = R_A * R_B$

or: $RR_{AB}^{orig} = R_{AB} / R_{b1} = RR_A^{orig} + RR_B^{orig} - 1 = (R_A + R_B) / R_{b1} - 1 = R_A + R_B - 1$

We can then define a new "*final*" reference population (*b2*) with a risk: $(R_{b2} \leq R_{b1})$

such that: $R_{b1} / R_{b2} = x \;; \quad x \geq 1$

In this case, relative to the new "*final*" reference population, the multiplicative relationship becomes:

$$RR_{AB}^{final} = RR_{AB}^{orig} * (x) \leq (RR_A^{orig} * RR_B^{orig}) * (x^2) = RR_A^{final} * RR_B^{final}$$

so that: $RR_{AB}^{final} \leq RR_A^{final} * RR_B^{final}$

and, similarly, the additive relationship becomes:

$$RR_{AB}^{final} = RR_{AB}^{orig} * (x) \leq (RR_{A}^{orig} + RR_{B}^{orig}) * (x) - 1 = RR_{A}^{final} + RR_{B}^{final} - 1$$

so that again: $RR_{AB}^{final} \leq RR_{A}^{final} + RR_{B}^{final} - 1$

Nevertheless, in the multiplicative model, the LHS of the inequality – i.e., (RR_{AB}^{final}) – increases by a factor of (x), whereas the RHS increases by a factor of (x^2). By contrast, in the additive model both sides increase (approximately) by the same factor of (x). Thus, as (x) increases, the disparity between the multiplicative model and what has actually been observed increases without bound, even in those circumstances where, using the "*original*" reference group, this model fits exactly. By contrast, for the additive model, as (x) increases, the disparity is much more modest and it reaches a limit at about a ~15% difference. Moreover, with only a modest reduction in the "*final*" compared to the "*original*" reference risk, the additive model provides a better fit than the multiplicative model, even in those circumstances where, using the "*original*" reference group, the multiplicative model fits exactly.

Consequently, it is important to consider which reference group (b) is the most appropriate to choose for calculating the *RRs*. Considering the theoretical underpinnings of these risk models [54-57], it is clear that the population with the least identifiable risk should be used as the reference group. As discussed elsewhere, there exists a large non-empty subset of the general population, the so-called (G–) subset, which has no risk of MS whatsoever [37-39]. Clearly, however, this subset cannot serve as a reference, because all *RRs* (using such a reference) would be either infinite or undefined. Indeed, the fact that, for MS, the (G–) subset is non-empty [37-39], indicates that both of these "risk models" are, at a theoretical level, invalid for characterizing the accumulation of disease risk with an increasing number of disease-associated "risk" factors.

Nevertheless, perhaps, a different reference group – i.e., one having, at least, some MS risk − could be used to evaluate (approximately) whether either of these two models fits with the available data. If so, because any notion of a "protective" genotype is relative, the most appropriate such reference would be the group having the smallest, non-zero, disease-risk of any. For example, in the present study, the smallest risk group we identified was for individuals with the (AP,AP) or ($AP*$) genotype (*see Methods, Main Text*). In the WTCCC, using this (AP^*) genotype as a reference, the ($0,0$) *MHC* genotype has an *RR* of:

$$(R_{(0,0)} / R_{AP*}) = RR_{(0,0)} \approx OR_{(0,0)} = 12.1$$

Therefore, to use the subset ($AP*$) as a reference group requires the magnitude of all

observed *RRs* (initially calculated with respect to the (*0,0*) *MHC* genotype) to be mathematically adjusted to reflect their relationship to (*AP**). Even having done this, however, there is no guarantee that the (*AP**) subset actually has the smallest, non-zero, MS-risk of any in the population. Indeed, because the (AP^*) subset specifies an individual's status only at the *MHC*, and considering that there are ~200 "MS-risk" loci in addition to the *MHC*, it seems likely that the (AP^*) subset will include some members with specific combinations of states at these other "risk" loci, which have an even lower (non-zero) MS-risk. This suggests that, for MS, one can never be confident that a multiplicative model is being supported, even approximately, by the data.

Table S1. Common Disease-associated *CEHs*, which include the (*H*+) Haplotype ††

Name†	(*H*+) *Haplotype* *A~C~B~DRB1~DQB1~SNP*	*OR (CI)**	Percentage*	p-value**
c2	*03:01~07:02~07:02~15:01~06:02~a1*	3.2 (3.0 – 3.5)	3.2%	< E-168
c3	*02:01~07:02~07:02~15:01~06:02~a1*	2.2 (2.0 – 2.5)	1.9%	< E-38
c6	*24:02~07:02~07:02~15:01~06:02~a1*	2.8 (2.4 – 3.3)	0.8%	< E-36
c11	*25:01~12:03~18:01~15:01~06:02~a1*	3.9 (3.1 – 4.8)	0.4%	< E-39
c13	*01:01~07:02~07:02~15:01~06:02~a1*	3.4 (2.7 – 4.2)	0.3%	< E-29
c16	*01:01~07:01~08:01~15:01~06:02~a1*	3.7 (2.9 – 4.8)	0.4%	< E-27
c19	*02:01~05:01~44:02~15:01~06:02~a1*	2.1 (1.6 – 2.7)	0.3%	< E-7
c22	*11:01~07:02~07:02~15:01~06:02~a1*	2.5 (1.9 – 3.4)	0.2%	< E-9
c28	*01:01~06:02~37:01~15:01~06:02~a1*	4.5 (3.2 – 6.3)	0.2%	< E-20
c44	*31:01~07:01~18:01~15:01~06:02~a1*	2.9 (2.0 – 4.2)	0.1%	< E-9
c50	*02:01~03:04~40:01~15:01~06:02~a1*	3.1 (2.0 – 4.7)	0.1%	< E-7
c58	*02:01~03:03~15:01~15:01~06:02~a1*	3.2 (2.1 – 5.0)	0.1%	< E-7
c78	*29:02~16:01~44:03~15:01~06:02~a1*	3.7 (2.2 – 6.1)	0.1%	< E-7
c87	*31:01~07:02~07:02~15:01~06:02~a1*	3.4 (2.0 – 5.6)	0.1%	< E-6
c91	*26:01~07:02~07:02~15:01~06:02~a1*	2.6 (1.6 – 4.3)	0.1%	< E-3
c108	*32:01~07:02~07:02~15:01~06:02~a1*	3.1 (1.8 – 5.4)	0.1%	< E-4
c116	*31:01~15:02~51:01~15:01~06:02~a1*	4.3 (2.4 – 7.9)	0.1%	< E-6
c120	*03:01~04:01~35:01~15:01~06:02~a1*	4.5 (2.5 – 8.1)	0.1%	< E-7
c128	*68:01~07:04~44:02~15:01~06:02~a1*	2.9 (1.6 – 5.1)	0.1%	< E-3
c139	*02:01~03:04~15:01~15:01~06:02~a1*	3.2 (1.6 – 6.3)	0.1%	< E-3
c140	*11:01~15:02~51:01~15:01~06:02~a1*	3.3 (1.7 – 6.4)	0.0%	< E-3
c143	*68:01~07:02~07:02~15:01~06:02~a1*	3.0 (1.6 – 5.6)	0.1%	< E-3
c173	*23:01~07:01~49:01~15:01~06:02~a1*	5.5 (2.8 –10.9)	0.0%	< E-7
c282	*03:01~15:02~51:01~15:01~06:02~a1*	20.3(6.1– 67.3)	0.0%	< E-11

†† *CEHs* carrying the (*H*+)-haplotype and having ≥ 50 representations in the WTCCC. Two other notable CEHs with fewer representations (*c173 & c282*) s are also shown. The (*H*+) haplotype indicates carriers of the *HLA-DRB1*15:01~HLA-DQB1*06:02~a1* motif.

† Arbitrary name for haplotype, sorted in descending order of frequency in the WTCCC [24,25].

* Odds ratio (OR) of disease for individuals having 1 copy of the listed haplotype compared to having no other copies of the (*H*+) haplotype. The 95% confidence interval (*CI*) is in parenthesis. Percentage indicates the % of all CEHs in the Control population.

** The p-values are expressed in scientific notation as powers of 10 (E).

Table S2. Other Common Disease-associated *CEH*s in the WTCCC Population†

Name††	*HLA Haplotype* *A~C~B~DRB1~DQB1~SNP*	*OR (CI)**	Percentage*	p-value**
c23	*30:02~05:01~18:01~03:01~02:01~a2*	2.0 (1.4 – 2.7)	0.3%	< E-4
c46	*01:01~07:01~08:01~03:01~02:01~a2*	2.1 (1.5 – 3.0)	0.2%	< E-4
c85	*02:01~05:01~18:01~03:01~02:01~a2*	1.7 (1.0 – 2.9)	0.1%	< 0.05
c1	*01:01~07:01~08:01~03:01~02:01~a6*	1.1 (1.0 – 1.2)	6.2%	< 0.05
c14	*02:01~07:01~08:01~03:01~02:01~a6*	0.9 (0.7 – 1.2)	0.7%	ns
c27	*03:01~07:01~08:01~03:01~02:01~a6*	1.7 (1.2 – 2.3)	0.3%	< E-2
c51	*68:01~07:01~08:01~03:01~02:01~a6*	0.6 (0.4 – 1.0)	0.3%	< 0.05
c68	*24:02~07:01~08:01~03:01~02:01~a6*	3.0 (1.8 – 4.9)	0.1%	< E-5
c90	*03:01~07:02~07:02~03:01~02:01~a6*	1.6 (0.9 – 2.6)	0.1%	ns
c97	*32:01~07:01~08:01~03:01~02:01~a6*	1.1 (0.6 – 2.0)	0.1%	ns
c110	*25:01~07:01~08:01~03:01~02:01~a6*	1.3 (0.7 – 2.3)	0.1%	ns
c34	*68:02~08:02~14:02~13:03~03:01~a14*	1.9 (1.3 – 2.8)	0.2%	< E-3
c96	*66:01~17:01~41:02~13:03~03:01~a14*	2.6 (1.5 – 4.5)	0.1%	< E-3
c107	*02:01~17:01~41:02~13:03~03:01~a14*	1.9 (1.1 – 3.4)	0.1%	< 0.05
c5	*02:01~05:01~44:02~04:01~03:01~a3*	0.5 (0.4 – 0.6)	1.9%	< E-11
c15	*02:01~06:02~13:02~07:01~02:02~a3*	0.5 (0.3 – 0.6)	0.7%	< E-5
c18	*02:01~06:02~57:01~07:01~03:03~a5*	0.5 (0.3 – 0.7)	0.6%	< E-4
c24	*02:01~01:02~27:05~01:01~05:01~a9*	0.5 (0.3 – 0.7)	0.5%	< E-3
c30	*02:01~05:01~44:02~11:01~03:01~a4*	0.6 (0.4 – 0.9)	0.4%	< 0.05
c32	*03:01~07:02~07:02~13:01~06:03~a18*	0.6 (0.4 – 0.9)	0.3%	< E-2
c73	*02:01~15:02~51:01~09:01~03:03~a4*	0.4 (0.2 – 0.8)	0.2%	< E-2
c81	*24:02~07:02~39:06~08:01~04:02~a16*	3.1 (1.8 – 5.5)	0.1%	< E-4

† *CEHs* not carrying the (*H*+) haplotype and having ≥ 50 representations in the WTCCC having a significant disease association. In addition, all such haplotypes carrying the *a2*, *a6*, or *a14* SNP haplotype are included.

†† Arbitrary name for haplotype, sorted in descending order of frequency in the WTCCC [24,25].

* Odds ratio (*OR*) of disease for individuals having 1 copy of the listed haplotype compared to having no copies of the particular *HLA-DRB1~HLA-DQB1~SNP* Class II haplotype being tested and also no copies of any (*H*+)-carrying *CEH* (e.g., *Table S2*). The 95% confidence interval (*CI*) is in parenthesis. Percentage indicates the % of all CEHs in the Control population.

** The p-values are expressed in scientific notation as powers of 10 (E); ns=not significant.

Table S3. Haplotype Group Frequencies in the WTCCC

Haplotype Group	1 copy	2 copies
H+	8,777	1,126
ER	1,036	12
AP	2,198	60
c1	3,640	161
0	12,155	14,770

Table S4a.† Percent overlap between the "most frequent" 1,000 *CEHs* in different world populations [25].*

	Af	Eu	Nai	Al	Mx	Ch	Jp	Ko
African (Af)	–							
European (Eu)	12.4	–						
N. Am. Indian (Nai)	12.3	59.3	–					
Aleut (Al)	8.0	28.8	31.6	–				
Mexican (Mx)	13.8	36.7	39.9	21.8	–			
Chinese (Ch)	2.8	7.8	7.6	4.5	5.3	–		
Japanese (Jp)	3.5	8.5	8.9	7.5	6.8	30.6	–	
Korean (Ko)	3.0	8.8	9.1	7.0	6.3	39.7	53.8	–
Asian Indian (Ai)	4.9	16.1	2.8	7.9	11.7	11.4	5.3	8.8

* Blue = <10% overlap
Yellow = 10 – 20% overlap
Red = >20% overlap

Table S4b.†† Percent overlap between "All African" *CEHs* and the "most frequent" 1,000 *CEHs* in different world populations [25].

	Eu	Nai	Al	Mx	Ch	Jp	Ko
All African	68.8	58.2	37.4	48.0	11.9	11.8	12.6

† In Table S3a the most common 1,000 *CEHs* in each population were determined and the percent overlap of these two sets of 1,000 *CEHs* is represented at the points of intersection for any two of the populations in the Table.

†† In Table S3b the row entries represent the percentage of the1,000 most frequent *CEHs* for each population that are found in the entire "*CEH set*" from the African population.

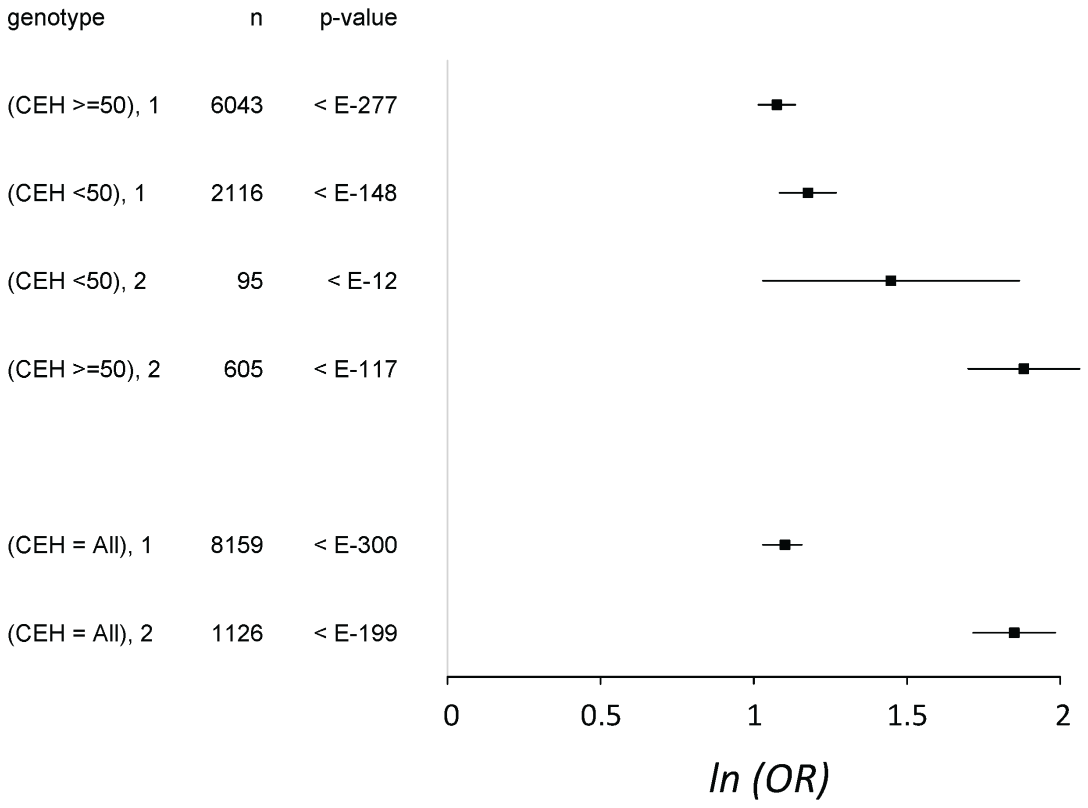

Supplemental Figure S1. Forest plots of the natural logarithm of the odds ratio (*OR*) of disease (together with the 95% Cis) for individuals having particular *CEH* combinations carrying the (*H+*)-motif compared to the (*0,0*) MHC genotype.

The *(H+)-motif = HLA-DRB1*15:01~HLA-DQB1*06:02~a1*.

(CEH <50), 1 = CEHs with 1 copy of (*H+*) and fewer than 50 representations in the WTCCC

(CEH <50), 2 = CEHs with 2 copies of (*H+*) and fewer than 50 representations in the WTCCC

(CEH ≥50), 1 = CEHs with 1 copy of (*H+*) and at least 50 representations in the WTCCC

(CEH ≥50), 2 = CEHs with 2 copies of (*H+*) and at least 50 representations in the WTCCC

(CEH =All), 1 = All *CEHs* with 1 copy of (*H+*) in the WTCCC

(CEH =All), 2 = All *CEHs* with 2 copies of (*H+*) in the WTCCC

p-values are expressed in scientific notation as powers of 10 (E); n=number of observations

ns=not significant.

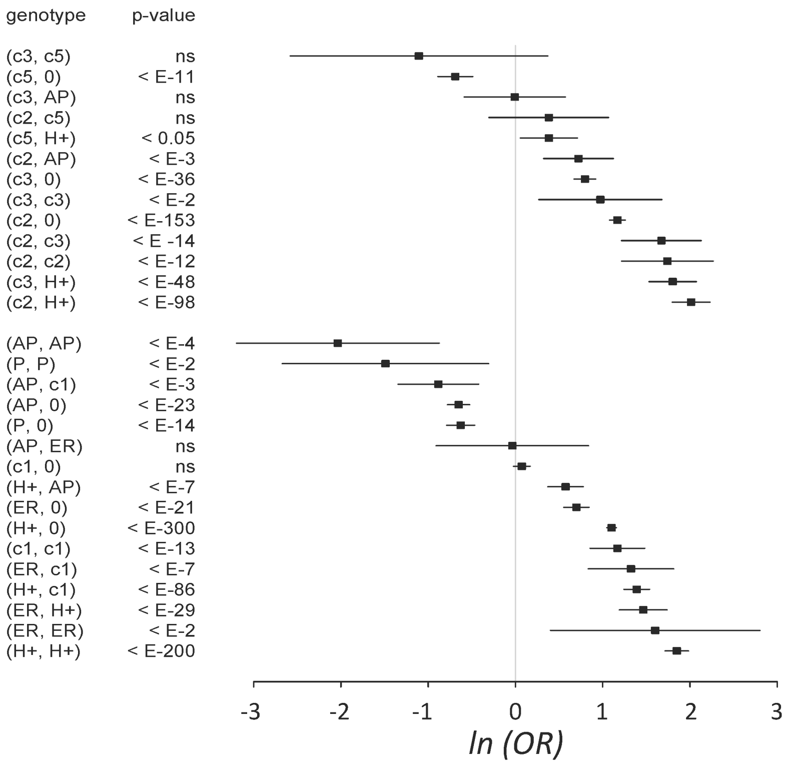

Supplemental Figure S2. Forest plots of the natural logarithm of the odds ratio (*OR*) of disease (with 95% CIs) for individuals having particular *CEH* genotypes compared to the (*0,0*) MHC genotype.

CEH names (*e.g., c1, c2, etc*) are defined in *Tables S1 & S2*).

Groups of CEHs are defined as:

*(H+)-motif = HLA-DRB1*15:01~HLA-DQB1*06:02~a1*

Protective (P); non-c5 = (c15, c18, c24, c30, c32, c51, and c73)

All Protective (AP) = (c5, c15, c18, c24, c30, c32, c51, and c73)

Extended Risk (ER) = (c23, c27, c34, c46, c68, c81, c85, c96, and c107)

Risk combinations were defined as:

"single copy risk"= 1 copy of any (H+) CEH or any (ER) CEH

and:

"double copy risk" = 2 copies of any (H+) CEHs, (c1), or 2 copies of any (ER) CEH or the combinations of (H+ and ER), (H+ and c1), and (ER and c1).

P-values are expressed in scientific notation as powers of 10 (E); ns=not significant.

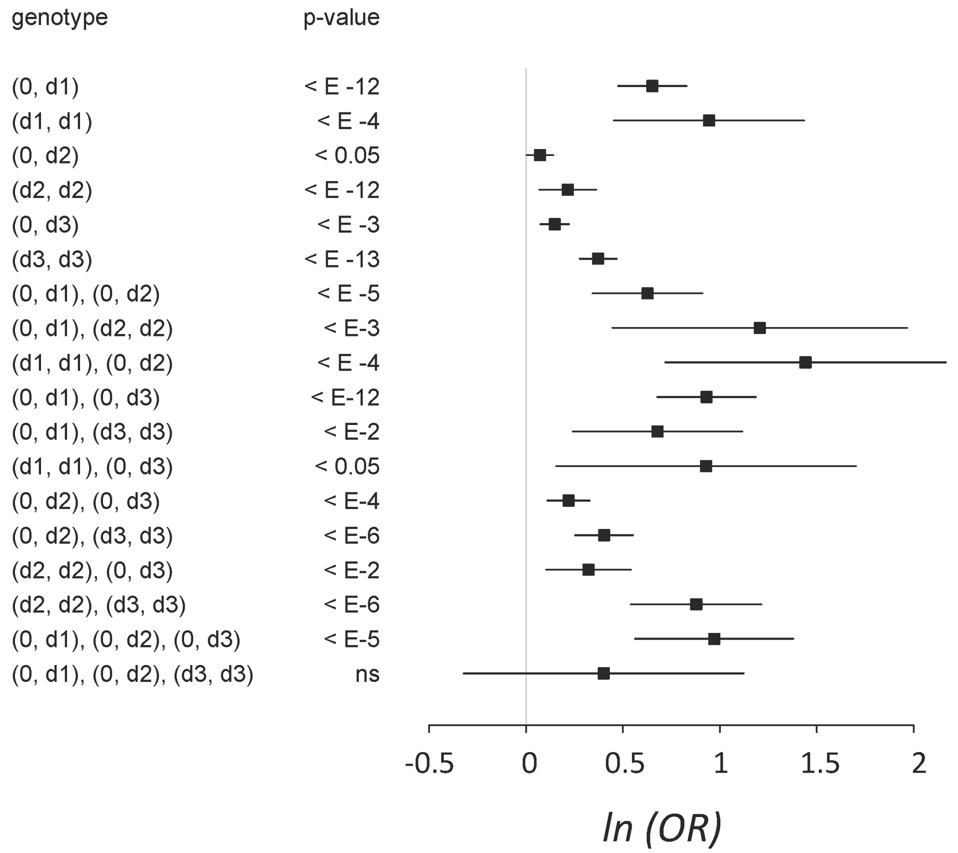

Supplemental Figure S3. Forest plot of the natural logarithm of the odds ratio (*OR*) of disease (with 95% CIs) for individuals having particular *non- MHC* genotypes compared to the (*0,0*) *MHC* genotype.

Risk haplotypes [24] for Non-MHC susceptibility loci (see text)

d1 = Region 22 in Chromosome 3 (EOMES)

d2 = Region 78 in Chromosome 14 (ZFP36L1)

d3 = Region 85 in Chromosome 16 (CLEC16A)

P-values are expressed in scientific notation as powers of 10 (E); ns=not significant.

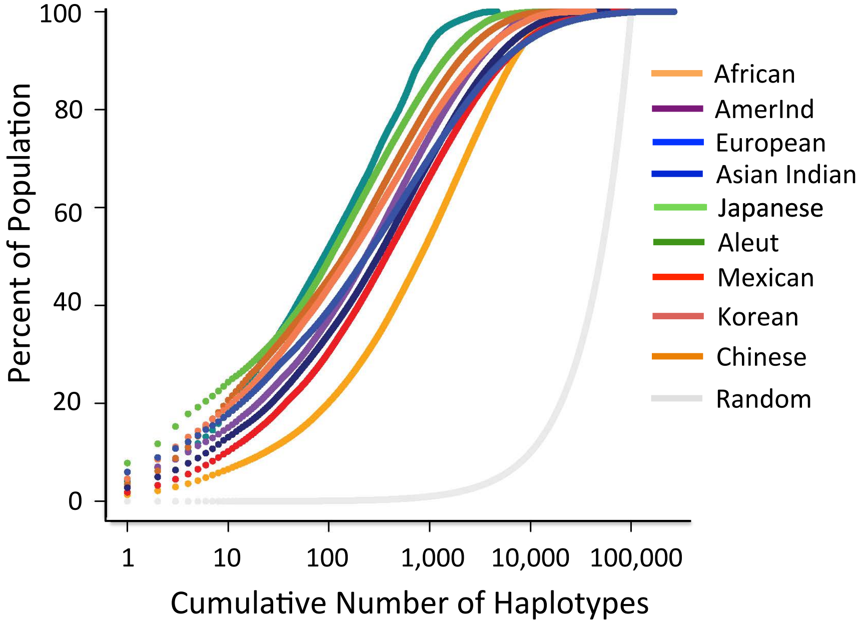

Supplemental Figure S4. Cumulative haplotype frequency distributions for *CEHs* (*A~C~B~DRB1~DQB1*) in different world populations [25]. *CEHs* were sorted in the order of descending haplotype frequency within each respective population. Also depicted (in grey) is the expected distribution if the *CEHs* were to be distributed randomly (i.e., un-selected). This graph plots the cumulative number of unique *CEH*s (beginning with the highest frequency CEH) in each population against the percentage of the total number of CEHs in that population. The large majority of the unique *CEHs* in all the different populations have only a small number of representations (low-frequency), whereas the majority of the total *CEHs* are accounted for in each population (including native Africans) by only a small number of high-frequency unique *CEHs*. The African population has greater *CEH* diversity than the other populations, presumably due to the fact that, when these other populations left Africa, they migrated with only a small selection of the available *CEHs*. Nevertheless, this pattern is true for every population and is markedly different from expectations for "randomly" distributed *CEHs* (grey line). Moreover, despite the fact that these populations derive from the same migration, the *CEHs* in the different populations have little overlap either with each other or with Africans. (*Supplemental Tables 3a & 3b*). Collectively, these observations indicate that this pattern must be due to selection, not to linkage disequilibrium. In fact, this selection pressure can be shown to be quite strong and that the survival of "novel" *CEHs* is only 54−79% compared to homozygous "non-novel" *CEHs* [26].
